# Avoiding incompatible drug pairs in central-venous catheters of patients receiving critical care: an algorithm-based analysis and a staff survey

**DOI:** 10.1007/s00228-023-03509-0

**Published:** 2023-06-07

**Authors:** Leonhardt Alexander Fabian Wagner, Martina Patrizia Neininger, Jan Hensen, Olaf Zube, Thilo Bertsche

**Affiliations:** 1 Bundeswehr Hospital, Hamburg, Germany; 2 Clinical Pharmacy, Institute of Pharmacy, Medical Faculty, Leipzig University, Bruederstraße 32, Leipzig, 04103 Germany; 3 Drug Safety Center, Leipzig University and Leipzig University Hospital, Bruederstraße 32, Leipzig, 04103 Germany

**Keywords:** Drug incompatibility, Critical care, Surveys and questionnaires, Knowledge, Algorithms

## Abstract

**Purpose:**

In a critical care setting, we aimed to identify and solve physico-chemical drug incompatibilities in central-venous catheters considering the staffs’ knowledge and assumptions about incompatibilities.

**Methods:**

(i) After positive ethical vote, an algorithm to identify incompatibilities was developed and applied. The algorithm was based on KIK^®^ database and Stabilis^®^ database, the drug label, and *Trissel* textbook. (ii) A questionnaire was created and used that asked staff for knowledge and assumptions about incompatibilities. (iii) A 4-step avoidance recommendation was developed and applied.

**Results:**

(i) At least one incompatibility was identified in 64 (61.4%) of 104 enrolled patients. Eighty one (62.3%) of 130 incompatible combinations affected piperacillin/tazobactam and in 18 (13.8%) each furosemide and pantoprazole. (ii) 37.8% (*n* = 14) of the staff members participated in the questionnaire survey (median age: 31, IQR: 4.75 years). The combination of piperacillin/tazobactam and pantoprazole was incorrectly judged to be compatible by 85.7%. Only rarely felt the majority of respondents unsafe in administering drugs (median score: 1; 0, never to 5, always). (iii) In those 64 patients with at least one incompatibility, 68 avoidance recommendations were given, and all were fully accepted. In 44 (64.7%) of 68 recommendations “Step 1: Administer sequentially” was suggested as a avoidance strategy. In 9/68 (13.2%) “Step 2: Use another lumen”, in 7/68 (10.3%) “Step 3: Take a break”, and in 8/68 (11.8%) “Step 4: Use catheters with more lumens” were recommended.

**Conclusions:**

Although incompatibilities were common, the staff rarely felt unsafe when administering drugs. Knowledge deficits correlated well with the incompatibilities identified. All recommendations were fully accepted.

**Supplementary Information:**

The online version contains supplementary material available at 10.1007/s00228-023-03509-0.

## Introduction

When drugs are used at the same time in the same intravenous lumen, physico-chemical incompatibilities can occur that affect patient safety [1–6]. The risk of incompatibilities increases when different IV drugs are used simultaneously. Incompatibilities are therefore of particular importance in critical care [7]. To avoid incompatibilities, therefore, information on incompatible drug pairs from literature has been compiled in databases. However, information from literature is often contradictory, and clear decisions are difficult to make whether a drug combination is compatible or not. Additionally, databases often lack practical recommendations for avoiding incompatibilities. What is more, causes of occurrence of incompatibilities such as knowledge deficits have hardly been addressed in publications so far [8].

To address all those gaps, we performed the present study addressing incompatibilities in central-venous catheters of patients receiving an interdisciplinary critical care. Here, we assessed drugs administered at the same time via a central-venous lumen for incompatibilities using a previously defined algorithm. The results of this analysis were compared to staff knowledge and assumptions about incompatibilities. Afterwards recommendation to avoid the identified incompatibilities was given, and their acceptance was evaluated.

## Methods

### Setting

The current study was conducted in a hospital characterized as a 300-bed military hospital providing tertiary care for civilian as well as for military patients. The study was performed in the interdisciplinary critical care unit of this hospital. No standards on incompatibilities had yet been introduced by the pharmacy in the setting. No survey had been performed so far to assess the level of knowledge of the staff on the topic of incompatibilities. At the time of the survey, no knowledge-based database was operating for incompatibilities and proposing recommendations to avoid incompatibilities. In the present setting, nutrition, sedation, and catecholamines were usually given separately. Regularly, 3-lumen central-venous catheters were used. For post-surgery patients, 5-lumen central-venous catheters were used. One lumen was used for catecholamines, another for analgesia and the last for further drugs (including total parenteral nutrition). For this purpose, several 3-lumen taps were strung together (sometimes up to 4–5 pieces). In the case of drugs for which an accidental administration of a bolus had relevant consequences for the patient (e.g., heparin, insulin), a peripheral venous catheter would be placed.

Short infusions and electrolytes were added to the feeding lumen; in 5-lumen central-venous catheters, these were usually administered by a separate catheter lumen. To be included in the analysis of compatible drug pairs, at least three drugs of a patient had to be given simultaneously via one lumen. Drugs from each catheter lumen were evaluated separately. The administration of the drugs through each lumen was documented in the patient chart.

### Study design

This was a monocentric, prospective study consisting of three study parts:

- **Incompatibility analysis** of central-venous administered drugs as documented in the patient chart (Part 1)
- **Questionnaire for a staff survey** to assess knowledge and assumptions about incompatibilities (Part 2)
- **Recommendations to avoid identified incompatibilities** as a four-step process including the evaluation of the acceptability (Part 3)

### Part 1: incompatibility analysis

**Fig. 1:**
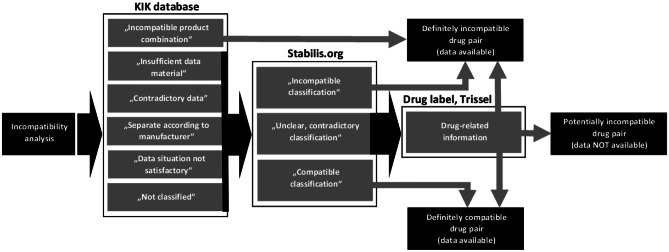
Procedure of an algorithm-based incompatibility analysis. The annotations in quotation marks correspond to what is given in the databases/sources

The drugs of consecutive patients on the first day of treatment were examined for incompatibilities in central-venous catheters using a predefined algorithm-based incompatibility analysis. The algorithm-based incompatibility analysis was used for the first time in a period from January 3, 2022, to April 8, 2022, where the drug charts of patients from the respective previous day, with the exception of weekends, were recorded every day from Monday to Friday. Within this period, all consecutive patients undergoing treatment in the critical care unit were recorded. The algorithm was designed as shown in Fig. 1, considering two databases available on the German market (KiK 4.0 compatibility in the catheter. Manufacturer: oData GmbH &Co. KG - Elbestraße 40 - 26180 Rastede and Stabilis database. French association without commercial goals. Available at infostab@stabilis.org. Last “update” on November 23, 2022), the summary of product characteristics, i.e., the drug label (preferably by the drug actually used or the original product), and Trissel LA: “Handbook of Injectable Drugs” American Society of Health-System Pharmacists; 17. Edition (October 31, 2012).

### Part 2: questionnaire for a staff survey incompatibilities

Staff consisting of physicians and nurses of the respective critical care unit were invited to answer questions on their knowledge and assumptions about incompatibilities in central-venous catheters by a questionnaire. The questionnaire was subjected to an independent pretest beforehand to improve the quality of the questionnaire. These data was not included in the main study.

After the project had been introduced in the unit meetings, the questionnaires were displayed in the break room of the setting, where they were accessible to the entire staff of the unit at all times. The staff members were asked to complete the questionnaire for themselves and not together with others. A contact person in the unit collected the anonymously completed questionnaires and handed them over to the study team in bundled form without personal reference. Care was taken in the questions on sociodemographic information to ensure that it could not be used to identify individuals.

The questionnaire consisted of the following parts:

#### Cover sheet

Addressing information about the study content and data protection (written informed consent).

#### Knowledge about incompatibilities

The staff was asked to indicate the compatibility of 11 drug pairs that were actually frequently administered via central-venous catheters in the unit. These were compatible or incompatible. Participants had to decide whether the combination was compatible or incompatible. It was then evaluated whether the answers were correct.

#### Assumptions about incompatibilities

The participants were asked to answer 15 questions about incompatibilities in central-venous catheters using the following Likert scale: 0, never; 1, rarely; 2, sometimes; 3, often; 4, mostly; and 5, always. It was also possible to tick “no answer possible”.

The correct answers were made available to the participants at the end of the study.

### Part 3: recommendations to avoid identified incompatibilities

**Fig. 2:**
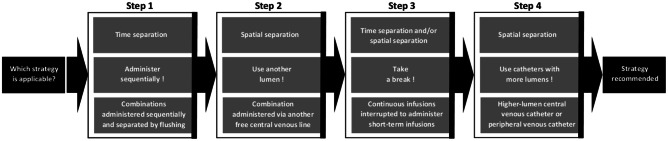
Strategies recommended to the staff of an interdisciplinary critical care unit to avoid incompatibilities identified by an algorithm-based incompatibility analysis in four steps. Presented are type of separation (upper box), strategies to avoid incompatibilities (middle box), and comments (lower box)

The incompatibility analysis included a predefined 4-step avoidance strategy (Fig. 2). The recommendations were forwarded to the staff involved in drug administration after the incompatibility analysis and the questionnaire survey had been finished. The acceptance of the recommendations by the staff was assessed.

### Statistical data analysis

Statistical analysis was performed using *Excel* 2019 *for Windows* and *IBM SPSS Statistics for Windows* (version 28, 2021). Data is presented as frequencies (in total numbers and in percent).

## Results

### Part 1: incompatibility analysis

#### Enrolled patients

Within the study period, 220 patients were admitted to the study unit. A total of 3297 drugs were administered during this period, from those 1710 (51.9%) central-venous drugs. A median of 15 (Q25/75 = 10/20) drugs per patient were administered, from those 7 (Q25/75 = 3/12) IV drugs. A total of 104 patients fulfilling the inclusion criteria (i.e., at least three drugs given simultaneously via one lumen) were enrolled in the incompatibility analysis. In those patients, 1976 drugs in total and 1234 central-venous drugs were administered. A median of 19 (Q25/75 = 15.75/23) drugs per patient were administered, of those 12 (Q25/75 = 8/15) central-venous drugs.

#### Usability of the algorithm-based analysis

For almost all combinations examined, the algorithm was able to make unambiguous assignments as to whether they were compatible or not. In 5 combinations, it was not possible to make a clear statement on the basis of algorithm: metamizole + vancomycin, metamizole + clindamycin, metamizole + metoclopramide, cefuroxime + urapidil, furosemide + glucose solution. These combinations were classified as not evaluable.

#### Identified incompatibilities

In the group of 104 patients, a total of 130 incompatible drug combinations were identified. For 64 (61.5%) of 104 patients at least one (median: 1.5, Q25/75; 0.5/2.5) physico-chemical incompatibility in central-venous catheters was found. Eighty one (62.3%) of 130 combinations involved anti-infectives such as antibiotics and antifungals. Furosemide and pantoprazole were affected in 18 (13.8%) of 130 combinations each, making them the most common combination partners of incompatible drug combinations (Table 1). The most common incompatible drug combinations occurred each 5 times and amounted to the combinations: erythromycin and metoclopramide, piperacillin/tazobactam and pantoprazole, prednisolone and ampicillin/sulbactam, cefuroxime, and Ringer’s acetate solution. Four times the combination of heparin and calcium chloride were identified. Three times each the combinations were found: erythromycin and multi-vitamin supplement (*Cernevit*^®^), metamizole and levetiracetam, piperacillin/tazobactam and ciprofloxacin, heparin and pantoprazole, piperacillin/tazobactam, and erythromycin. From the group of the most frequent incompatible drug combinations, an active substance from anti-infectives occurred at least once in *n* = 7. A cross-table of all identified incompatible combinations is shown in Table 2.

**Table 1:** Frequencies of drugs involved in incompatible drug combinations in an incompatibility analysis in an interdisciplinary critical care unit (*n* = 130 drug combinations)

**Drug**	**Frequency [*n*]**	**Percent [%]**
Furosemide	18	13.8
Pantoprazole	18	13.8
Piperacillin/tazobactam	17	13.1
Heparin	16	12.3
Erythromycin	16	12.3
Cefuroxime	11	8.4
Ringer’s acetate	11	8.4
Ampicillin/sulbactam	10	7.7
Metoclopramide	9	6.9
Caspofungin	8	6.2
Dipyrone	8	6.2
Levetiracetam	7	5.4
Prednisolone	6	4.6
Magnesium sulfate	6	4.6
Metronidazole	6	4.6
Meropenem	6	4.6
Ciprofloxacin	6	4.6
Amiodarone	6	4.6
Linezolid	5	3.8
Calcium chloride	5	3.8

**Table 2:**
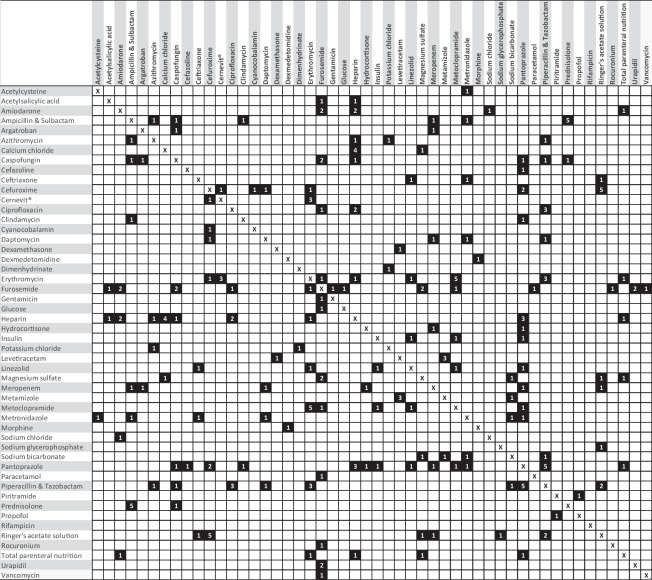
Cross-table of definitely incompatible drug pairs (data available) identified by an algorithm-based incompatibility analysis

The figures refer to the number of drug pairs observed

### Part 2: knowledge and assumptions about incompatibilities

#### Enrolled staff members

Of the total staff of 37 in the critical care unit, from those 30 nurses and 7 physicians, 14 participated in the questionnaire survey, from those 11 nurses, 2 physicians, and one without further specification. Participants’ age ranged from 23 to 45 years, with a median age of 31 (Q25/Q75: 26.25/35.75) years. Median work experience was 9 (Q25/Q75: 4.5/13.5) years.

#### Knowledge and assumptions survey

In a total of 154 responses to the knowledge questions, 103 responses were rated as correct, 49 as incorrect, and 2 as not answered (Table 3). The assumptions about incompatibilities in different items are shown in Table 4.

**Table 3:** Knowledge of staff members of the critical care unit in a questionnaire about compatible or incompatible drug combination pairs that occur frequently in this unit (*n* = 14 participants with a total of 154 responses)

**Drug #1**	**Drug #2**	**Compatible**	**Correct answer**	**Incorrect answer**	**No answer**
Amiodarone	Calcium chloride	No	100%	0%	0%
Esketamine	Dexmedetomidine	Yes	79%	21%	0%
Sufentanil	Adrenaline	Yes	57%	43%	0%
Amiodarone	Sodium chloride	No	100%	0%	0%
Propofol	Esketamine	Yes	93%	7%	0%
**Piperacillin/tazobactam**	**Pantoprazole**	No	14%	86%	0%
Heparine	Potassium chloride	Yes	21%	79%	0%
Cefuroxime	Sodium bicarbonate	No	79%	7%	14%
Nutrition (Smofkabiven)	Metronidazole	Yes	79%	21%	0%
Nutrition (Smofkabiven)	Cefuroxime	Yes	71%	29%	0%
**Furosemide**	**Pantoprazole**	No	43%	57%	0%

Bold background: The three drugs most frequently identified as incompatible in the medical record analysis

**Table 4:** Assumptions of staff members of the critical care unit in a questionnaire according to the following categories: 0, never; 1, rarely; 2, sometimes; 3, often; 4, mostly; and 5, always

**Question**	**Median**	**Q25**	**Q75**	**Min**	**Max**
1. How often is more than one drug administered via the same catheter line?	4	3	4	2	5
2. How often do you feel unsafe while administering drugs?	1	1	2	1	4
3. How often do you not feel confident while administering multiple medications in succession?	1	1	2	1	2
4. How often do you think physico-chemical problems occur in your department?	1	1	1	1	3
5. How often do you think these problems have serious consequences?	1	1	2	1	2
6. How often do you feel that two drugs should better not be administered together because of incompatibilities?	2	1	2	1	2
7. Nevertheless, how often do you administer them?	1	0.25	1	0	3
8. How often do you contact your nursing staff colleagues with any questions regarding incompatibilities?	2.5	2	5	1	5
9. How often do you contact your medical staff colleagues with any questions regarding incompatibilities?	3	2	4	1	5
10. How often do you search drug information sources regarding incompatibilities, e.g., the summary of product characteristics?	2	1	2.75	0	5
11. How often do you not feel confident in reconstituting?	1	1	1.75	1	2
12. How often do you contact your nursing staff colleagues if you have any questions regarding the solvent/carrier solution?	3.5	2	4	1	5
13. How often do you contact your medical staff colleagues if you have any questions regarding the solvent/carrier solution?	2	2	3	0	5
14. How often do you search drug information sources regarding solvent/carrier solution, e.g., the summary of product characteristics?	2.5	1.25	3.75	1	5
15. How often do you deal with questions regarding compatibility or physico-chemical stability of drugs outside of working hours?	1	0.25	1	0	2

“No answer” was possible (*n* = 14 participants)

### Part 3: recommendations to avoid identified incompatibilities

In 64 patients with at least one incompatible drug combinations, recommendations were given according to a 4-step procedure to the staff to avoid the identified incompatibilities (Fig. 2). In 4 of these patients, two different recommendations of the four steps were made simultaneously, and in 60, a single recommendation was made resulting in a total of 68 recommendations (1.08 per patient).

Of the avoidance strategies, 44 (64.7%) of 68 involved a successive administration of the incompatible combinations “Administer sequentially” in Step 1. For 9 (13.2%) of 68, the avoidance strategy “Use another lumen” was recommended in Step 2. Another 7 (10.3%) of 68 recommendations contained the solution strategy “Take a break” in Step 3. In the remaining 8 (11.8%) of 68 recommendations, it was advised to use catheters with more lumens in Step 4. All recommendations (*n* = 68, 100%) were judged to be useful for implementation in practice by the staff involved in routine central-venous drug administration.

## Discussion

### General considerations

Incompatibilities jeopardize patient safety, especially in critical care, due to multiple central-venous drug combinations. Using an algorithm-based incompatibility analysis, incompatibilities were identified in 61.4% of 104 enrolled patients. Furosemide, pantoprazole, and piperacillin/tazobactam were particularly commonly involved in incompatible drug pairs. In a corresponding questionnaire survey, combinations with piperacillin/tazobactam as well as with pantoprazole were incorrectly considered compatible by the majority, explaining the incompatibilities actually found. Only rarely did the majority of respondents feel unsafe when administering drugs. Sequential administration of the incompatible combinations was recommended as the most common avoidance strategy. All recommended measures were fully accepted by those performing routine central-venous drug administration.

### Methodical aspects

To date, few studies have examined a comprehensive arsenal of databases and other sources of incompatibilities. A study [9] that did address this issue showed different levels of completeness and accuracy. The authors recommended the development of standards for assessing incompatibilities as we put into practice by the current study. An advantage of our study is therefore that we developed and applied an algorithm that can also be used to evaluate conflicting findings from different databases as reported also by others [10]. This represents a further development and no longer places the burden of this difficult decision-making on the individual decision maker on the single ward. The 4-step concept for the preparation of recommendations for the avoidance of identified incompatibilities is a further special feature of the present study. As reported by others [3], we additionally developed a cross-table to give an overview over the identified incompatible drug pairs but added specific recommendations for individual identification of incompatibilities. Other strategies to avoid incompatibilities recommended in literature are an extensive training for staff administering central-venous drugs or the creation of in-house standards [11]. Such measures also prove useful from the results of the present study. In particular, the congruence of the knowledge deficits with the incompatibilities actually identified shows that it makes sense to provide information about these incompatibilities, for example, in the form of teaching sessions or internal standards.

Additional volumes were recommended with caution or not at all in the case of patients with salt and fluid restrictions. On the other hand, a larger common volume in extension sets of tap distributors compared to short multi-lumen lines was not considered particularly as those aspects were performed by the nursing staff, and our influence on these measures was limited. However, this would certainly a useful measure for future considerations. For the diluent used for irrigation, only solutions appropriate for the respective drugs were recommended or those that were explicitly listed in databases as compatible.

### Incompatibilities as a frequent problem

Results from Tissot et al. [7] showed that about 14% of all medication errors in a critical care setting were caused by incompatibilities, while Taxis et al. [12] even attributed up to 25% of all medication errors to incompatibilities. In [3], incompatibilities were found for 12% of drug pairs infused in an critical care setting. Together with the results obtained here, it is clear that physico-chemical incompatibilities remain a frequent hazard for patients. In relation to the total number of all our patients (*n* = 220), there was a high number of incompatible drug pairs (i.e., 29%). In relation to the selection of patients who met the inclusion criteria and had at least three drugs administered equally via a lumen, the number was even higher: the number of incompatible drug combinations affected 61.5% of the patients who suffered from at least one incompatibility. In a direct comparison with data from Roveda Marsilio et al. (68%) [13] and Moraes et al. (78.5%) [14], our rate seems to comparable high. We found an average of 8.8 drugs prescribed per patient, while the mean number of intravenously administered drugs was 7 per patient in [14]. If the number of prescribed central-venous drugs increases, the estimated risk for potential incompatibility reactions increases substantially [15].

### Drugs frequently affected by incompatibilities

#### Pantoprazole

According to our results, among others, pantoprazole was most frequently represented in incompatibilities. In [3], pantoprazole as well has been shown the drug most frequently involved. Comparable results, especially with regard to pantoprazole, were also found in a previous survey in pediatric critical care [16]. The fact that pantoprazole emerges from the data as one of the most frequently problematic drugs is due to the fact that in the hospital studied, pantoprazole is used almost exclusively instead of omeprazole or esomprazole. Pantoprazole is probably prescribed more frequently because compared to omeprazole it has a lower drug-drug interaction potential [17].

#### Furosemide

Furosemide is used as a continuous infusion in many patients. This means that many of the incompatibility reactions occur because a long-term infusion of furosemide is already at the catheter lumen, although other drugs need to be administered via a short infusion or single dose. The slow flow rates of such a long-term infusion contribute, through prolonged contact times, to the development of possible incompatibility reactions. Another reason for a frequent involvement of furosemide in incompatibilities is that it is a loop diuretic that belongs to a frequently administered group of agents.

#### Piperacillin/tazobactam

Piperacillin/tazobactam belongs to a group of anti-infectives that were the most frequently drugs involved in incompatibilities in our study. Out of 130 drug combinations identified as “incompatible”, 62.3% contained at least one drug from anti-infectives. The reason for this high share of involvement of anti-infectives in incompatibilities is their urgency of central-venous route of administration in critical care patients. For many preparations, constant administration is required on identical times in order to ensure constant active ingredient plasma levels. When comparing the data of Moraes et al. [14] and Neininger et al. [10] with the data in the present study, it becomes apparent that piperacillin/tazobactam do not represent outliers, but can frequently be found in the literature. Neininger et al. [10] postulated that vancomycin is one of the drugs most frequently involved in incompatible combinations, whereas Moraes et al. [14] mentioned, among others, piperacillin/tazobactam most frequently.

### Evidence of drug incompatibilities

An interesting point concerns the evidence of the information of incompatibilities. In the databases used within this study, the evidence was not explicitly stated. Frequently, the existence of corresponding studies of an analytical nature was already the only source of information. As a rule, data were not distinguished by static or real-time compatibility studies, drug concentrations, proportions, diluents, or contact times. Due to the lack of data, the level of evidence of compatibility data was not reviewed in this study. However, evidence should become an important issue in this area as well. For example, we stated in this study that piperacillin/tazobactam and pantoprazole are incompatible. However, this assessment was only based on one paper without a high level of evidence in the Stabilis^®^ database. Additionally, no specification of the drug concentrations was available [18]. What is more, for the drug incompatibility furosemide and pantoprazole, evidence was based on only two papers [18, 19]. One of the papers does not note a drug incompatibility between pantoprazole and furosemide. The other paper does not specify the drug concentrations, and the results are doubtful. These examples show that the database for assessing evidence for incompatibilities is often incomplete. Often, no clinical data are available at all, or influencing factors are not sufficiently specified. This is mainly because this topic is not as much in the focus of clinical research as, for example, drug-drug interactions. Nevertheless, with relatively limited resources for studies on incompatibilities, the commitment of such authors should not be underestimated.

### Strategies to avoid incompatibilities

In addition to analyzing the various problematic drug combinations and creating a cross-tabulation table as frequently reported in literature, this paper also offered tailored avoidance strategies in each affected patient. A four-step process was used to escalate the avoidance strategies as required. This was done to limit the effort required for avoidance measures. The goal was to avoid low-practice recommendations, such as administering drugs through ever-new separate lines, whenever possible or to perform them only when other options were not feasible. Of the 4 avoidance strategies, the first step, which involved successive administration of the incompatible combinations one after the other, was already recommended in 64.7% of the recommendations to avoid incompatibilities. The 100% acceptance of all recommendations underlines the meaningfulness in the eyes of the professional groups involved in the evaluation, i.e., physicians and nurses.

“Step 1: Administer sequentially”, “Step 2: Use another lumen”, “Step 3: Take a break”, and “Step 4: Use catheters with more lumens” were recommended steps to avoid identified drug incompatibilities according to our algorithm. This way, we had deliberately chosen a 4-step procedure. Switching to peroral dosage forms might be an interesting idea, but was out of the question in the context of this study, as, e.g., only medical reasons were considered acceptable by the treatment team for sequential antibiotic therapy. What is more, we did not consider the use of diluted drug infusions to be a sufficiently reliable strategy.

### Limitations

The following limitations should be considered:

- *First*, the results refer only to one critical care setting of one hospital.
- *Second*, neither clinical consequences of the incompatibilities nor a follow-up of the recommended and accepted avoidance strategies were analyzed.
- *Third*, although it is highly unlikely that the incompatibilities would have been eliminated during the course of treatment without the recommendations, we do not know this with absolute certainty since this study was performed as a before-after comparison without a control group.
- *Fourth*, we focused on incompatibilities of central-venous drugs—particularly relevant in our setting. However, incompatibilities are also possible in peripheral-venous catheters and mixing bags.
- *Fifth*, the time while applying the algorithm was not assessed. If the algorithm will be used in a real emergency situation, it should be considered that a certain time is required to find an appropriate combination by checking three data sources according to this algorithm.
- *Sixth*, even if the staff was asked to complete the questionnaire for themselves, it cannot be ruled out that questionnaires were filled out in groups in single cases, even though there were no indications of this in the evaluation.
- *Seventh*, no visual compatibility tests of drug mixtures were performed in the unit to account for drug mixture concentrations and proportions (e.g., depending on the mass flow rate of the drugs).
- *Eighth*, prescription patterns were not examined in detail. However, description such as proximal lumen, medial lumen, and distal lumen was not explicitly addressed in the prescriptions.

## Conclusion

Although incompatibilities in central-venous administered drugs were common, staff in the corresponding critical care unit rarely felt unsafe when administering drugs. Knowledge deficits correlated well with incompatibilities identified. All recommended 4-step measures were fully accepted by the staff.

## Supplementary Information

Below is the link to the electronic supplementary material.

Supplementary file1 (DOCX 18 KB)

## Data Availability

The data can be requested for scientific questions from the corresponding author.
