# Supplementary material for: Avoiding incompatible drug pairs in central-venous catheters of patients receiving critical care: an algorithm-based analysis and a staff survey
Source: Eur J Clin Pharmacol. 2023 Jun 7;79(8):1081–9. doi: 10.1007/s00228-023-03509-0 (PMC10361869; doi:10.1007/s00228-023-03509-0)
Supplement: Supplementary file 1 — Supplementary file1 (DOCX 18 KB) [file 228_2023_3509_MOESM1_ESM.docx]

**Supplementary Material**

**Examples of specific recommendations and relevant drugs in recommendation Steps 1-4:**

***Recommendations Step 1:*** ***Administer sequentially !***

- 6 different i.v. drugs; incompatible combination: piperacillin/tazobactam and pantoprazole: the duration of the antibiotics infusion was 4 hours and after 3 hours of the current administration, pantoprazole was added. This infusion could also have been administered one hour later.
- 6 different i.v. medications; incompatible combination: ampicillin/sulbactam and prednisolone: the duration of the antibiotics infusion lasted several hours and prednisolone was applied about halfway through; since no lumen was clear and an emergency could not be assumed from the remaining medication, the later administration was recommended.
- 5 different i.v. drugs; incompatible combination: Ringer's acetate solution and piperacillin/tazobactam: due to the pharmacological urgency of the administration of Ringer's acetate solution, which can be considered rather low, it is quite possible to wait for the complete administration of the antibiotics.
- 7 different i.v. drugs; incompatible combination: piperacillin/tazobactam and erythromycin: the temporal urgency or the stricter temporal administration regime of beta-lactam antibiotics requires a strict administration of piperacillin/tazobactam, which may well be followed by the administration of erythromycin (as a temporally less sensitive antibiotic).
- 4 different i.v. drugs; incompatible combination: aspirin and heparin: aspirin as an on-demand medication does not have to be given during the administration of the pharmacotherapeutic more relevant heparin, but can follow the administration after irrigation.

***Recommendations Step 2:*** ***Use another lumen !***

- 16 different i.v. drugs; incompatible combination: urapidil and heparin, pantoprazole and heparin, pantoprazole and linezolid, ceftriaxone and linezolid: here, due to the quite considerable number of incompatible combinations, sequential administration was not suggested, but the possibility of using a different lumen. At the given times, another lumen was free in each case, so that both heparin and linezolid could have been applied to different lumens in each case. In addition, heparin can be administered s.c., which would have left only one lumen free.
- 6 different i.v. drugs; incompatible combination: urapidil and furosemide: at the given time one lumen was free, considering the fact that furosemide was administered as a short infusion, a change of the lumen was therefore possible without problems
- 8 different i.v. drugs; incompatible combination: urapidil and furosemide, urapidil and metamizole: sequential administration should not be the solution here, since one lumen was unused and both urapidil and metamizole should be used immediately as demand medication in acute cases.
- 12 different i.v. drugs; incompatible combination: a total parenteral nutrition and erythromycin: the permanent infusion of a total parenteral nutrition as well as the infusion of erythromycin cannot be administered sequentially and should also not be interrupted for longer periods of time, since another lumen was still free at this time, switching to another lumen is an option.
- 8 different i.v. drugs; incompatible combination: pantoprazole and a total parenteral nutrition, Pantoprazole and Cefazolin: a temporal separation is not possible due to the permanent administration of a total parenteral nutrition, since another lumen is also available, the use of this lumen for the administration of pantoprazole is obvious.

***Recommendations Step 3******: Take a break !***

- 6 different i.v. drugs; incompatible combination: calcium chloride and heparin: during the continuous infusion of heparin, no other lumen was free at any time, which is why use of another lumen was omitted. The interruption of the continuous heparin infusion should not be a major problem due to the low flow rate of the continuous infusion and can easily be interrupted for a short infusion.
- 12 different i.v. drugs; incompatible combination: heparin and calcium chloride, heparin and cyanocobalamin: analogous to the example given. Cyanocobalamin must be administered separately in addition, which is why the interruption of heparin administration is also necessary here.
- 9 different i.v. drugs; incompatible combination: heparin and caspofungin: analogous to the above examples.

***Recommendations Step 4:*** ***Use catheters with more lumens !***

- 16 different i.v. drugs; incompatible combination: 9 incompatible combinations: the sheer number of incompatible combinations made a separate administration both temporally and spatially impossible via a free lumen. To avoid the most relevant incompatibilities, it is necessary to change the central venous catheter to a larger model, possibly the administration of a peripheral venous catheter is additionally necessary.
